# Doxorubicin resistant cancer cells activate myeloid-derived suppressor cells by releasing PGE_2_

**DOI:** 10.1038/srep23824

**Published:** 2016-04-01

**Authors:** Yuan Rong, Chun-Hui Yuan, Zhen Qu, Hu Zhou, Qing Guan, Na Yang, Xiao-Hua Leng, Lang Bu, Ke Wu, Fu-Bing Wang

**Affiliations:** 1 Department of Laboratory Medicine & Center for Gene Diagnosis, Zhongnan Hospital of Wuhan University, No. 169 Donghu Road, Wuchang District, Wuhan 430071, P.R. China; 2 Department of Immunology, School of Basic Medical Sciences, Wuhan University, No. 185 Donghu Road, Wuchang District, Wuhan 430071, P.R. China; 3 Hubei Key Laboratory of Tumor Biological Behaviors & Hubei Cancer Clinical Study Center, Zhongnan Hospital of Wuhan University, No. 169 Donghu Road, Wuchang District, Wuhan 430071, P.R. China; 4 Department of Pathophysiology, School of Basic Medical Sciences, Wuhan University, No. 185 Donghu Road, Wuchang District, Wuhan 430071, P.R. China; 5 Animal experiment center of wuhan university/Animal Biosafety Level-III laboratory, No. 115 Donghu Road, Wuchang District, Wuhan 430071, P.R. China

## Abstract

Chemotherapies often induce drug-resistance in cancer cells and simultaneously stimulate proliferation and activation of Myeloid-Derived Suppressor Cells (MDSCs) to inhibit anti-tumor T cells, thus result in poor prognosis of patients with breast cancers. To date, the mechanism underlying the expansion of MDSCs in response to chemotherapies is poorly understood. In the present study, we used *in vitro* cell culture and *in vivo* animal studies to demonstrate that doxorubicin-resistant breast cancer cells secret significantly more prostaglandin E_2_ (PGE_2_) than their parental doxorubicin-sensitive cells. The secreted PGE_2_ can stimulate expansion and polymerization of MDSCs by directly target to its receptors, EP2/EP4, on the surface of MDSCs, which consequently triggers production of miR-10a through activating PKA signaling. More importantly, activated MDSCs can inhibit CD4^+^CD25^−^ T cells as evidenced by reduced proliferation and IFN-γ release. In order to determine the molecular pathway that involves miR-10a mediated activation of MDSCs, biochemical and pharmacological studies were carried out. We found that miR-10a can activate AMPK signaling to promote expansion and activation of MDSCs. Thus, these results reveal, for the first time, a novel role of PGE_2_/miR-10a/AMPK signaling axis in chemotherapy-induced immune resistance, which might be targeted for treatment of chemotherapy resistant tumors.

Myeloid-derived suppressor cells (MDSCs) are a heterogeneous population of immature myeloid cells that are substantially expanded in various disease states including cancer, and are capable of supporting tumor growth through remodeling of the tumor microenvironment^1^,^2^. MDSCs are functionally characterized by their T-cell-suppressive activity via arginase 1 (Arg 1), like promoting the generation of regulatory T (Treg) cells, and phenotypically, murine MDSCs are characterized by expression of Gr1 and CD11b cell surface markers^3^,^4^. Recently, multiple reports have indicated that Tumor-induced MDSCs expansion significantly contributes to the mechanisms of cancer-induced immune suppression, thus different approaches have been explored to target the functional crosstalk between tumor cells and the MDSCs^5^,^6^. Chemotherapy drugs, like doxorubicin, have been broadly used in the treatment of variety of cancers, such as breast cancer. While it could selectively eliminated MDSCs that accumulated in breast tumor microenvironment, the killing effects of doxorubicin on MDSCs are transient and these cells eventually will be recurrent^7^. This may be own to that doxorubicin-induced CXCL1/2 expression in treated tumor cells could attract more CD11b^+^Gr1^+^ MDSCs into the tumor microenvironment^8^ and soluble factors that secreted by treated tumor cells, like TGF-β, can be recognized and trigger miRNAs expression in MDSCs and thus promote proliferation and activation of tumor-expanded MDSCs^9^,^10^,^11^.

Prostaglandin E_2_ (PGE_2_) is a factor released by tumor cells undergoing programmed apoptotic death as a result of chemotherapy. It has been identified as endogenous lipid mediator involving angiogenesis and immune tolerance^12^. The immune-modulatory effects of PGE_2_ on MDSCs are largely due to its ability to induce the upregulation of the expression of M2 marker Arg 1 through PGE_2_ receptors EP2-EP4 and the downstream effector cAMP-PKA cascade^13^,^14^. Accordingly, the inhibitors of COX2, which is required for the production of PGE_2_, were found to improve anti-tumor T-cell responses by downregulating the ARG1 expression of MDSCs^15^,^16^.

MicroRNAs are endogenous, non-coding RNAs of approximately 22 nucleotides that target various genes via translational repression or target mRNA degradation^17^,^18^. Recent studies have shown that miRNAs, like miR-620 and miR-17–92, are able to modulate the sensitivity of cancer cells to chemotherapeutic drugs and therefore contribute to the acquisition of chemo-resistance^19^,^20^. Furthermore, aberrant upregulated expression of miRNAs, like miR-21, miR-494, miR-155, contributes to MDSCs expansion and thereby suppressing local immunity and limiting the efficacy of various chemotherapies^10^,^11^. In addition, increased levels of miR-10a have been found in many cancers, including primary hepatocellular carcinomas, breast cancer and glioblastoma^21^,^22^,^23^. However, it remains largely unknown whether miR-10a is responsible for regulation of MDSCs in tumor microenvironments. Furthermore, although recent findings suggest that miR-10a may regulate resistance to chemotherapies^22^,^24^, an exact mechanism of miR-10a dependent acquired immune resistance following chemotherapy had not yet to be elucidated. Thus, it is urgent to further understand the functional crosstalk between doxorubicin-resistant tumor cells and MDSCs, which could be critical for designing more effective therapies to overcome resistance and improve outcome of cancer patients.

Here we demonstrated, for the first time, that PGE_2_ secreted by doxorubicin-resistant breast cancer 4T1 cells enhanced the expansion and M2 polarization of MDSCs via upregulating miR-10a expression in MDSCs. miR-10a inhibitor treatment abrogated the elevated frequency of CD11b^+^Gr-1^+^ cells, namely MDSCs, which induced by PGE_2_, and also the expression of M2 signature genes, such as Arg1, MMP9 and TGF-β. Furthermore, miR-10a inhibitor partially abolished the PGE_2_-induced inhibitory actions of MDSCs on proliferation and IFN-γ production of CD4^+^ T cells. Further biochemical and pharmacological experiments revealed that AMPK is a downstream factor in response of upregulation of miR-10a for activation of MDSCs. Taken together, these findings suggested molecular mechanism underlying doxorubicin induced inhibition of tumor immunity which might be a potential target for treatment of chemotherapy resistant cancers.

## Results

### DOX-resistant breast cancer cells induces the miR-10a expression and functional MDSCs expansion

**Figure 1:**
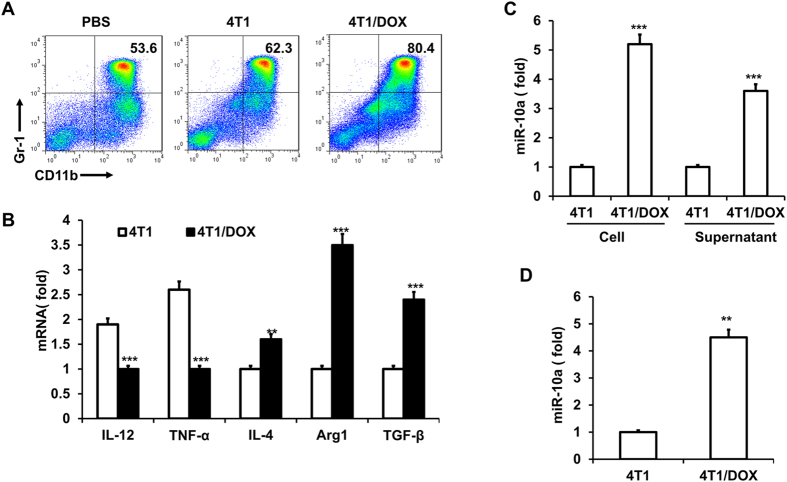
Doxorubicin-resistant tumor cells induce miR-10a expression and functional MDSCs expansion. Bone marrow (BM) cells were cultured with PBS or conditioned medium from 4T1 or Doxorubicin-resistant 4T1 cells (4T1/DOX) for 4 days in the presence of GM-CSF and IL-6, the frequency of MDSCs in the BM cells was analyzed by flowcytometry (**A**), MDSCs were sorted by FACS and genes expression of MDSCs were analyzed by qRT-PCR (**B**). (**C**) BM cells were cultured with 4T1 cells and 4T1/DOX cells or their derived conditioned medium; in the presence of GM-CSF and IL-6 for 24 h, and the expression of miR-10a in MDSCs was measured by qRT-PCR. (**D**) The expression of miR-10a in MDSCs sorted from spleen two weeks after 4T1 or 4T1/DOX cells were intravenously inoculated into BALB/c mice through the tail vein. The data represent Mean ± SD, (n = 5). **p < 0.01, ***p < 0.0001 means 4T1/DOX group *vs.* 4T1 group.

Immuno-resistance is one of the major obstacles in chemotherapy of breast cancer patients. To mimic the chemotherapy protocol applied in the clinics, we repeatedly treated the murine mammary carcinoma 4T1 with doxorubicin for several cycles *in vitro*^25^,^26^. After fourth treatment, 4T1 cells acquired chemo-resistant phenotype to doxorubicin (4T1/DOX). Because accumulating evidence demonstrates that tumor cells with drug resistant are responsible for rendering MDSCs with the ability to promote immune resistance, we thus examined the role of DOX-resistant 4T1 cells (4T1/DOX) for the differentiation of MDSCs in tumor microenvironments. We cultured bone marrow (BM) cells with the conditioned medium (supernatant) from 4T1/DOX cells (without DOX) or parental 4T1 cells in the presence of GM-CSF and IL-6. We found that treatment of BM cells with supernatant from 4T1/DOX cells resulted in a marked increase of CD11b^+^Gr-1^+^ MDSCs as compared to that from parental 4T1 cells (Fig. 1A). MDSCs can further be characterized into monocytic (M-MDSC, Gr-1^Low^ly6G^−^Ly6C^High^CD115^+^) and granulocytic (G-MDSC, GR-1^High^Ly6G^+^Ly6C^Low^CD115^−^) subsets^27^,^28^,^29^. Our data showed both of M-MDSCs and G-MDSCs were greatly increased after the treatment of supernatant from 4T1/DOX cells (Supplementary Fig. S1). Moreover, MDSCs sorted from BM cells that treated with supernatant from 4T1/DOX cells showed higher expression of the M2 markers Arg1, IL-4, and TGF-β, whereas the expression of the M1 markers TNF-α and IL-12 was significantly decreased as compared with that treated with 4T1 control supernatant (Fig. 1B). Thus, these results indicate that a soluble factor(s) derived from DOX-resistant tumor cells induce the expansion of MDSCs and preferentially promote the polarization of MDSCs into M2-type phenotypes.

Given the accumulating evidences indicate that microRNAs control MDSCs expansion and polarization in basal conditions, we hypothesized that aberrant expression of miRNAs might contribute to MDSCs expansion under chemotherapeutic conditions. To this end, we directly co-cultured BM cells with either 4T1 cells or 4T1/DOX cells and found co-culture of 4T1/DOX cells will trigger highly elevated expression of four miRNAs and downregulated expression of three miRNAs in MDSCs as compared to those cultured with parental 4T1 cells (Supplementary Fig. S2). Among these miRNAs, miR-10a had the highest upregulation after co-cultured induction (Supplementary Fig. S2). This result was further validated by qRT-PCR analysis (Fig. 1C). This is also true when we culture MDSCs with conditioned medium derived from 4T1/DOX cells (Fig. 1C), indicating a soluble factor(s) from 4T1/DOX might be responsible for upregulation of miR-10a in MDSCs. As expected, co-culture of 4T1/DOX cells significantly increased expression of miR-10a in both subpopulations of MDSCs compared with those from co-culture of parental 4T1 cells (Supplementary Fig. S3). Furthermore, to investigate whether DOX-resistant 4T1 cells lead to the enhanced expression of miR-10a of MDSCs *in vivo*, we inoculated parental 4T1 cells or 4T1/DOX cells into BALB/c mice through tail-vein injection and examined the expression of miR-10a of MDSCs in the spleen two weeks later. In consistent with the observation *in vitro*, we found the expression of miR-10a was significantly elevated in MDSCs isolated from 4T1/DOX tumor-bearing mice as compared to those from 4T1 tumor-bearing mice (Fig. 1D). Indeed, 4T1 breast tumor-bearing mice were repeatedly treated with DOX (5 mg/kg) at 15 days post-tumor implantation. We also found the expression of miR-10a was significantly elevated in MDSCs isolated from 4T1 tumor-bearing mice systemically treated with DOX as compared to those from PBS-treated mice as a control (Supplementary Fig. S4). Taken together, these results demonstrated that DOX-resistant tumor cells promote expansion and M2-type polarization of MDSCs both *in vitro* and *in vivo* through releasing a soluble factor(s).

### DOX-resistant tumor cells exploit PGE_2_ to regulate miR-10a expression in MDSCs

**Figure 2:**
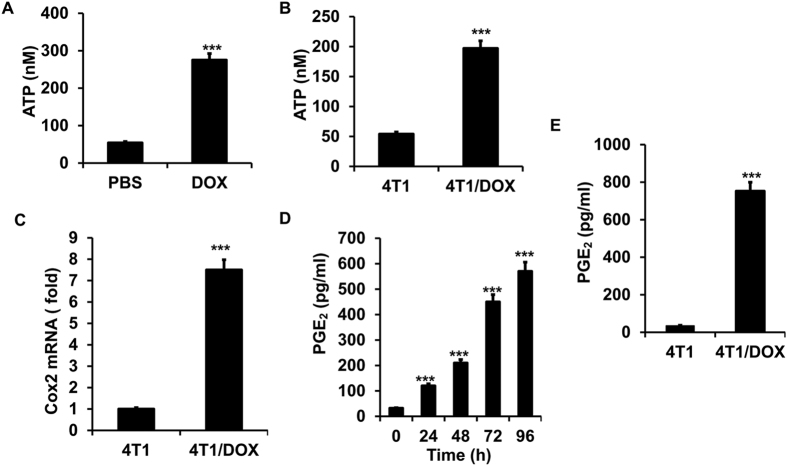
Doxorubicin induces the release of PGE_2_ from tumor cells. (**A**) 4T1 cells was treated with PBS or 50 nM doxorubicin for 72 hours. Extracellular ATP was examined. (**B**) The production of extracellular ATP in 4T1 or Doxorubicin-resistant 4T1 cells (4T1/DOX) were also examined. (**C**) The expression of COX2 in 4T1 or Doxorubicin-resistant 4T1 cells (4T1/DOX) was examined. (**D**) 4T1 cells was treated with 50 nM doxorubicin for different hours, and the level of extracellular PGE_2_ was examined. (**E**) The level of extracellular PGE_2_ in 4T1 or 4T1/DOX cells was examined. The data represent Mean ± SD. (n = 5).

In order to search for the secreted factor(s) from doxorubicin-resistant 4T1 cells that is responsible for activation of MDSCs. We found that chronic exposure to doxorubicin induced high amount of ATP release of 4T1 cells compared to that of cells incubated in doxorubicin-free media (Fig. 2A), indicating the enzymes for ATP production might be altered in 4T1/DOX cells. Indeed, ATP production was greatly increased in 4T1/DOX cells as compared to that in parental 4T1 cells (Fig. 2B). qRT-PCR analysis of genes commonly associated with ATP production revealed that cyclooxygenase-2 (COX-2) upregulated for more than 6-fold in 4T1/DOX cells as compared to that in parental 4T1 cells (Fig. 2C). As COX-2 and its downstream products such as PGE_2_ have widespread immune-modulatory roles in the development of tumor-associated suppressive macrophages and MDSCs^30^,^31^, we then investigated the PGE_2_ level after doxorubicin treatment and observed a significant enhanced production of PGE_2_ in 4T1 cells with chronic exposure to 50 nM doxorubicin in a time dependent manner (Fig. 2D). Indeed, the supernatant from 4T1/DOX contained higher level of PGE_2_ as compared to that from parental 4T1 cells (Fig. 2E).

**Figure 3:**
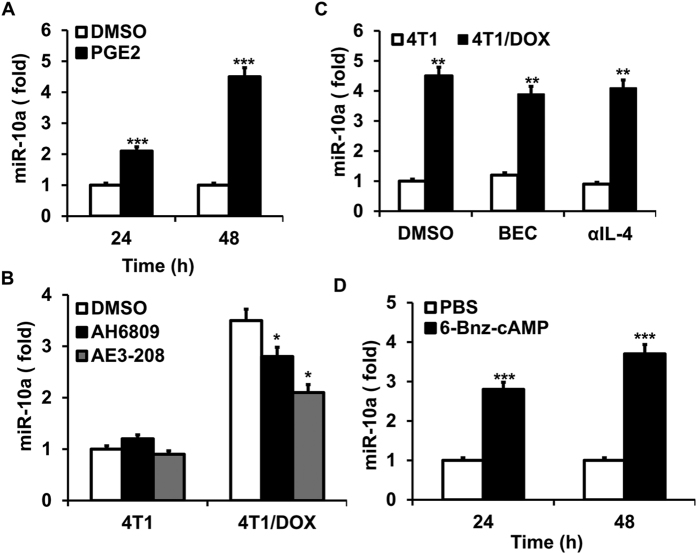
PGE_2_ induces miR-10a expression in MDSCs via the cAMP/PKA signaling pathway. MDSCs were treated with DMSO control or 100 μM PGE_2_ (**A**); 500 μM of PKA-specific cAMP analog 6-Bnz-cAMP (**D**) in the presence of GM-CSF and IL-6 for the indicated times, and the expression of miR-10a was determined by qRT-PCR. BM cells were cultured with conditioned medium supernatant of 4T1 cells or 4T1/DOX cells in combination with/without EP4 (1 μM ONO-AE3-208) or EP2 (AH6809) antagonist (**B**), arginase inhibitor S-(2-boronoethyl)-L-cysteine (BEC) and anti-IL-4 neutralizing Ab (αIL-4) (**C**), as well as in the presence of GM-CSF and IL-6 for 24 h, and the expression of miR-10a in sorted MDSCs was measured by qRT-PCR. Data represent Mean ± SD from 3–5 individual experiments. *p < 0.05, **p < 0.01, ***p < 0.0001 means *vs*. DMSO or PBS control.

It has been demonstrated that PGE_2_ stimulates a panel of microRNAs and regulates macrophage polarization in a PKA dependent manner^32^,^33^, however, whether PGE_2_ and its downstream microRNAs play a role on regulation of MDSCs is poorly understood. To examine whether PGE_2_ regulates miR-10a expression in MDSCs, bone marrow cells were treated with PGE_2_ for 24 h and 48 h. We found that miR-10a expression was upregulated when MDSCs treated with recombinant PGE_2_ at both time points (Fig. 3A). In addition, the immune-modulatory effects of PGE_2_ in MDSCs largely result from its ability to increase intracellular cAMP through binding to its receptors EP2 and EP4 34. Indeed, when we treated MDSCs with AH6809 (an EP2-selective antagonist) and ONO-AE3-208 (an EP4-selective antagonist), we can partially inhibit induction of miR-10a expression by supernatant of 4T1/ODX (Fig. 3B). Interestingly, the inhibitors for IL-4 and arginase-1, which are critical for M2 MDSCs differentiation and functional activities^35^,^36^, had little effect on 4T1/DOX-induced miR-10a expression of MDSCs (Fig. 3C). To further confirm the role of PKA on induction of miR-10a, we treated MDSCs with 6-Bnz-cAMP, agonist of PKA (cAMP downstream effector), and found that treatment of 6-Bnz-cAMP induced the expression of miR-10a in MDSCs (Fig. 3D). Taken together, these results suggest that PGE_2_ secreted by DOX-resistant tumor cells activates EP2-EP4/ cAMP/PKA signaling pathway in MDSCs and subsequently induces miR-10a expression.

### MiR-10a is an endogenous stimulator in PGE_2_-mediated immune suppression in MDSCs

**Figure 4:**
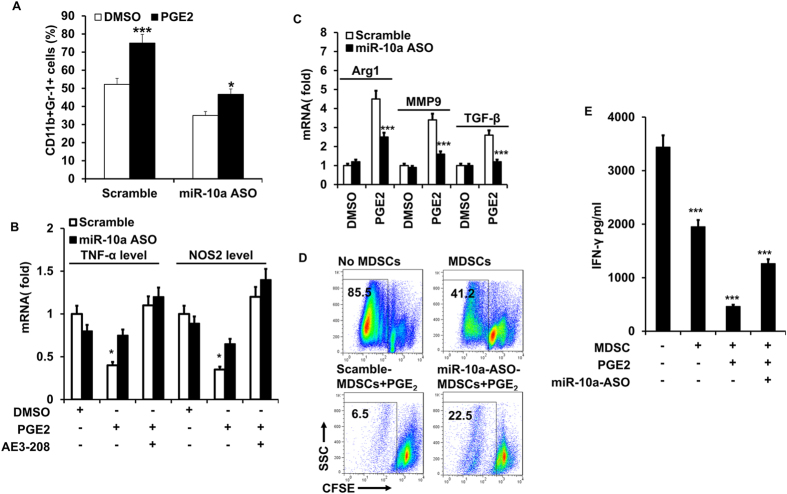
PGE_2_ promotes the expansion and M2 polarization of MDSCs via miR-10a. BM cells were treated with PGE_2_ in the presence of GM-CSF and IL-6. MiR-10a antagomir (miR-10a ASO) and scrambled oligonucleotides were transfected on the second day. Gr-1^+^CD11b^+^ MDSCs were evaluated by flowcytometry after 4 days (**A**). BM cells in (**A**) were cultured with (**B**) or without (**C**) EP4 antagonist (1 μM ONO-AE3-208), rhe relative levels of TNF-α, NOS2, Arg1, MMP9, and TGF-β mRNA in BM-derived MDSCs were detected by qRT-PCR after 3 days of transfection; (**D**) *In vitro* suppressive ability of MDSCs that transfected with miR-10a antagomir (miR-10a ASO) or scrambled control on naïve CD4^+^CD25^−^ T cells proliferation were analyzed by flowcytometry. CFSE labeled CD4^+^CD25^−^ naïve T cells were incubated with APC (CD4^+^ T cell depleted splenocytes) and MDSCs, and stimulated with anti-CD3 with/without PGE_2_ for 3–5 days. Cell proliferation was measured as a function of CFSE dilution. (**E**) The level of IFN-γ in the supernatant of cocultured cells in D was determined by ELISA. Data represent Mean ± SD from 3–5 individual experiments. *p < 0.05, **p < 0.01, ***p < 0.0001 means vs control.

To characterize the effect of miR-10a on immunomodulation function of MDSCs, BM cells were transfected with miR-10a inhibitor for 5 days in the presence or absence of PGE_2_. As shown in Fig. 4A, PGE_2_ increased the frequency of MDSCs among BM cells cultured with GM-CSF and IL-6, whereas miR-10a inhibitor reduces the induction of MDSCs by PGE_2_. To study the role of miR-10a in polarization of PGE_2_-stimulated MDSCs, BM cells transfected with miR-10a inhibitor were challenged with PGE_2_ in the presence and absence of EP4 antagonist. PGE_2_ significantly decreased the expression of M1 markers TNF-α and NOS2 (Fig. 4B) and enhanced the expression of M2 markers Arg1, MMP9, and TGF-β (Fig. 4C) in MDSCs that sorted from BM cells transfected with scramble oligos, however, treatment of miR-10a inhibitor significantly attenuated the effects of PGE_2_ (Fig. 4C). Furthermore, addition of EP4 antagonist ONO-AE3-208 significantly increased M1 genes expression in MDSCs, while no effect on M1 genes expression was observed in miR-10a knockdown MDSCs as compared to scramble control (Fig. 4B). Next, we sought to determine whether miR-10a could enhance the immunomodulation functions of MDSCs by measuring their function in inhibiting T cells proliferation. CD4^+^CD25^−^ naïve cells were labeled with carboxyfluorescein succinimidyl ester (CFSE), a fluorescence of which decreases proportionally as cells proliferate (Fig. 4D, No MDSCs). Addition of MDSCs suppresses cell division and the secretion of IFN-γ (Fig. 4D,E) and PGE_2_ further enhanced the suppressive ability (Fig. 4D,E), while miR-10a knockdown partially reversed suppressive capacity of MDSCs that enhanced with the presence of PGE_2_ (Fig. 4D,E). These data suggest that miR-10a acts as an endogenous stimulator of PGE_2_ to upregulate the expansion and polarization of MDSCs, as well as enhance suppressive capacity on CD4 T cells.

### PGE_2_ enhances M2 polarization of MDSCs via activating AMPK signaling

**Figure 5:**
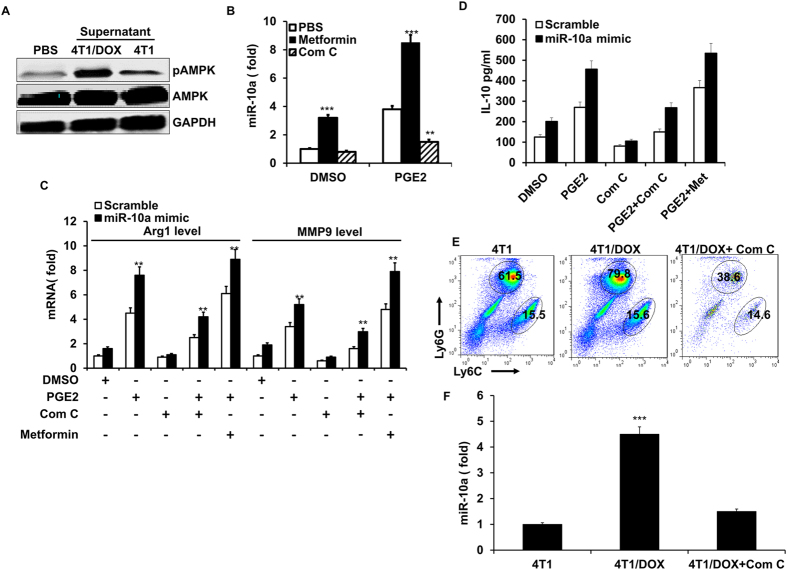
The role of AMPK in the miR-10a expression of MDSCs induced by PGE2. (**A**) Western blotting of p-AMPK and AMPK in BM-derived MDSCs cultured with conditioned medium from 4T1 or Doxorubicin-resistant 4T1 cells (4T1/DOX) for 3 days in the presence of GM-CSF and IL-6. (**B**) BM-derived MDSCs transfected with miR-10a antagomir (miR-10a ASO) or scrambled control were cultured with metformin (10mM) or compound C (Com C, 5 μM) in the presence of or absence of PGE_2_, and the expression of miR-10a was determined by qRT-PCR. (**C**) BM-derived MDSCs transfected with miR-10a mimics or scrambled control were cultured with metformin (10 mM) or Compound C (Com C, 5 μM) in the presence of or absence of PGE_2_, MMP9 and Arg1 mRNA were determined by qRT-PCR. (**D**) MDSCs were cultured as C and activated by LPS (100 ng/ml) and IFN-γ (2 ng/ml), the secretion of IL-10 was examined by ELISA. (**E**) Representative FACS plots showing the percentage of M-MDSCs and G-MDSCs (cells were gated on CD11b^+^Gr-1^+^) in spleen two weeks after 4T1/DOX cells were intravenously inoculated into BALB/c mice with/without Compound C treatment (25 mg/kg, one time/every three days for four times). (**F**) Quantification of the expression of miR-10 in CD11b^+^Gr-1^+^ MDSCs sorted from spleen. Data represent Mean ± SD from 3 individual experiments. **p < 0.01, ***p < 0.0001 means vs control.

The release of ATP to the tumor supernatant suggested that the cellular AMP-to-ATP ratio was higher. The heterotrimeric kinase, AMPK, is activated by intracellular energy deficits brought about by increased AMP-to-ATP ratio when the cell’s energy state is low^37^, indicating AMPK may be implicated in the regulation of myeloid cells differentiation during chemotherapy process^38^. Indeed, analysis of western blot showed the expression level of phosphorylated AMPK (p-AMPK) was significantly increased in the MDSCs cultured with supernatant derived from 4T1/DOX cells compared to that treated with supernatant from parental 4T1 cells in the presence of GM-CSF and IL-6 (Fig. 5A). Since AMPK is critical to PGE_2_-induced differentiation of BM cells to endothelial progenitor cells^39^ and suppresses the secretion of a T cell stimulating factor IL-12p40 40. Furthermore, a recent study identified that IL-12/IL-23p40 serve as a target of miR-10a in dendritic cells^41^, thus we hypothesized that AMPK might play a role in PGE_2_-mediated miR-10a expression in MDSCs. In order to test the potential role of AMPK on miR-10a mediated activation of MDSCs, we assessed the levels of miR-10a in MDSCs treated with AMPK activator, metformin in the presence of PGE_2_. As shown in Fig. 5B, metformin treatment enhanced the levels of miR-10a in MDSCs induced by PGE_2;_ however, the treatment of AMPK inhibitor, compound C, greatly suppressed the expression of miR-10a induced by PGE_2_. Furthermore, while overexpression of miR-10a significantly increased the mRNA levels of M2 markers, Arg1 and MMP9, in MDSCs, the treatment of metformin or compound C, greatly enhanced or attenuated the expression of M2 markers respectively (Fig. 5C). In addition, metformin or compound C has the similar role in the production of immunosuppressive cytokine IL-10 by MDSCs activated with LPS and IFN-γ when miR-10a was overexpressed (Fig. 5D); supporting the hypothesis that AMPK promotes the MDSCs polarization following upregulation of miR-10a. These findings provided a compelling rationale for assessing compound C in a therapeutic setting. Thus, we evaluated compound C for efficacy in blocking the development of MDSCs *in vivo*. Administration of compound C concurrent with 4T1/DOX cells implantation significantly inhibited the accumulation of G-MDSCs, but not M-MDSCs in spleen (Fig. 5E). We also found the expression of miR-10a was significantly decreased in MDSCs isolated from 4T1/DOX tumor-bearing mice systemically treated with Compound C as compared to those from vehicle-treated mice as a control (Fig. 5F).

**Figure 6:**
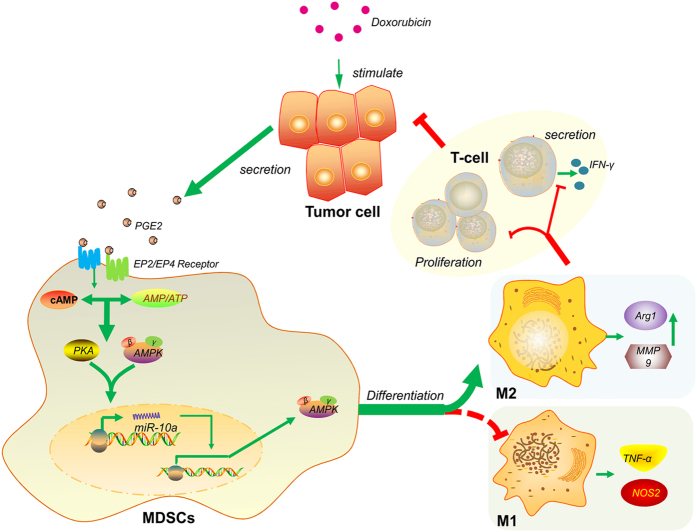
A schematic summary of the findings in this study to demonstrate doxorubicin induced defect of anti-tumor immunity. Doxorubicin treatment leads to PGE_2_ release from cancer cells. PGE_2_ can target its receptors, EP2/EP4 receptors, on the surface of MDSCs to activate PKA and AMPK signaling, induce production of miR-10a and enhance AMPK signaling in MDSCs. This signaling cascade turns on expansion and polarization of MDSCs for their inhibitory action on T cells, which are responsible for anti-cancer immunity.

Taken together, as shown in Fig. 6, we propose a novel cellular and molecular mechanism underlying chemotherapy induced anti-cancer immunity. We found that DOX treatment enhances PGE_2_ secretion from tumor cells, which target to the EP2/EP4 receptors on the surface of MDSCs for their activation. The binding of PGE_2_ with EP2/EP4 receptors activates PKA signaling and upregulates expression of miR-10a, which promotes expansion and polarization of MDSCs through activation of AMPK. Thus, targeting this process might be an effective means for treatment of chemo-resistant cancer patients.

## Discussion

In humans, chemotherapy-driven MDSCs expansion has been observed in cancer patients and appears to correlate with increased metastatic tumor burden^42^. Previous studies have reported that tumor-induced inflammation can recruit and expand MDSCs capable of suppressing antitumor immune responses^43^,^44^. Although the tumor-promoting effects of chronic inflammation has been well established^45^, the impacts of chemotherapy-induced inflammation or resistance on the recruitment of MDSCs has not been fully elaborated. In this study, we mimicked the general chemotherapy protocol of breast cancer treatment in *in vitro* cell culture model by treating the epithelial breast cancer cell line 4T1 sequentially in four cycles with doxorubicin treatment^25^,^26^, which generated a DOX resistant cell line 4T1/DOX. The fact that the supernatant derived from drug-resistant 4T1 tumor cells can drive the expansion of immunosuppressive MDSCs suggests that a soluble factor(s) produced by drug-resistant cells may elicit myeloid suppressor cells that counteract the ultimate efficacy of chemotherapy. It has been shown that doxorubicin induces an inflammatory immune milieu, in which myeloid growth factors and chemotactic factors such as GM-CSF, PGE_2_, and CCL2 are abundant^46^,^47^. PGE_2_ has been found to promote MDSCs recruitment to the tumor microenvironment through the induction of CXCL12 chemokine and lead to an upregulation of ARG1 expression, which accordingly regulates MDSCs-related T-cell immune suppression^48^. Veltman *et al*.^49^ also demonstrated that COX2 inhibition with dietary celecoxib treatment improved immunotherapy and prevented the local and systemic expansion of all MDSC subtypes in a mesothelioma murine model. We showed that doxorubicin-resistant 4T1 tumor cells upregulated the mRNA expression of COX2 and markedly enhanced PGE_2_ secretion; which suggest that simultaneous blockage of the PGE_2_ and COX2 loop may provide a potential target for the recruitment and differentiation of MDSCs^35^.

MicroRNAs (miRNAs) have attracted considerable attention in mediating the induction and expansion of MDSCs^50^. For example, miR-223 51, miR-21 and miR-20a^52^ alleviated the immunosuppressive potential of MDSCs by targeting MEF2C and STAT3 expression, respectively; whereas miR-155 and miR-21a may be associated with regulating the accumulation and functions of tumor-expanded MDSCs via targeting PTEN, a tumor suppressor gene^53^. Here, we showed that co-culture of 4T1/DOX breast cancer cells or its derived supernatant with MDSCs leads to increased miR-10a levels and an altered M2 phenotype of MDSCs. Although the role of miR-10a in regulation of epithelial-mesenchymal transition (EMT) are well investigated and established^21^,^54^, an exact mechanism of miR-10a-mediated immune resistance in MDSCs remains unknown. This study highlights a novel regulatory mechanism of MDSCs expansion along the PGE_2_/cAMP/PKA axis mediated by miR-10a. Firstly, we investigated the role of the cAMP inducer PGE_2_ in the expression of miR-10a in MDSCs. Our data show that PGE_2_ stimulation significantly enhanced miR-10a expression, and incubation of MDSCs with the downstream effector of cAMP, PKA agonist also increased miR-10a levels. The heterogeneous effects of PGE_2_ are reflected by the existence of four different PGE_2_ receptors, EP2 and EP4 receptors are the main receptors involved in the induction of MDSCs^55^. We found that AH6809-treated and ONO-AE3-208-treated MDSCs have partially reduced the level of miR-10a in the presence of PGE_2_, which indicating the EP2-EP4 receptor is the triggers for the expression of miR-10a in MDSCs, and other receptors like EP3 may also involve in PGE_2_-induced miR-10a expression^56^. Our data demonstrate that miR-10a is the main component that enhanced MDSCs expansion, as the miR-10a inhibitor significantly decreased MDSCs expansion even with the presence of PGE_2_, which alone could efficiently induce high levels of Gr1^+^CD11b^+^ cells that differentiated from bone marrow precursor cells. These data lead us to speculate that miR-10a acts as an effector that PGE_2_ exploited to exert immunosuppressive effects.

There is evidence that doxorubicin treatment inhibits cellular respiration in a number of *in vitro* models resulting in a decline in cellular ATP levels and resultant toxicity^57^,^58^. This change in ATP release will decrease the ratio of ATP-to-ADP. AMPK is a major cellular energy sensor and is sensitive to the cellular ratio of AMP to ATP^59^. A high AMP or low ATP level activates AMPK, which inhibits energy-consuming processes and enhances energy-producing processes to restore the energy homeostasis^60^. PGE_2_ exerts its action through the two Gs coupled receptors, EP2 and EP4, is mediated by the adenylate cyclase-triggered cAMP/PKA pathway. EP_4_ receptor subtype can trigger a signaling paradigm that promotes AMPK activation^61^. Therefore, PGE_2_ may regulate AMPK activity via activating PKA signaling pathway, which needs to be investigated in our future study. Recent study also showed AMPK is implicated in the differentiation of BM cells to MDSCs^38^. Stimulation of macrophages with anti-inflammatory cytokines such as IL-10, IL-4, and TGF-β results in rapid activation of AMPK, suggesting that AMPK contributes to the accumulation of M2 MDSCs. M2 MDSCs accelerates tumor growth mainly by enhanced immunosuppression involving an increase in arginase and immunosuppressive cytokines. In recent years, the effect of PGE2 on MDSCs accumulation and suppressive function was found to be mediated by STAT3^36^,^62^. STAT3 targeting can affect MDSCs expansion and differentiation and lead to differentiation of MDSCs by miRNAs, like miR-21, miR-181b and miR-155 53. The possible molecular mechanisms underlying whether PGE2/AMPK regulates STAT3-activated miRNAs will be interesting areas for future experiments. Here, we investigated the crosstalk between PGE_2_ and AMPK in the regulation of miR-10a expression during the induction of MDSCs, and confirmed that AMPK synergistically cooperated with PGE_2_ to enhance miR-10a expression and serve as a downstream molecule of miR-10a to promote MDSCs toward a M2 phenotype, as reflected by the enhanced expression of MMP9 and Arg1. Importantly, our data showed that administration of compound C significantly inhibits the accumulation of G-MDSCs in spleen from drug-resistant tumor bearing mice. However, AMPK also have been shown to suppress the proliferation and migration of cancer cells through modulating the expression of miRNAs, including miR-21, miR-106, let-7 and miR-181^63^,^64^, which suggest that AMPK activation may exert divergent roles in the progress of tumor development. An improved understanding of the mechanisms underlying these observations may allow us to optimize the use of anticancer drug to prevent or limit inflammation-related tumor progression. Therefore, the potential role of AMPK/STAT3 via miR-10a in modulating the myeloid-cell inflammatory response through MDSCs and M2 macrophages should be closely explored.

Doxorubicin is widely used in chemotherapeutic regimen primarily for its conventional direct tumoricidal activity, however, to our knowledge, the data from this study provide the first evidence that PGE_2_ secreted by Doxorubicin-resistant tumor cells can promote the expansion and polarization of MDSCs via upregulating their endogenous miR-10a expression, and the activation of cAMP/PKA and EP4/AMPK signaling pathway play pivotal roles in this activity.

## Materials and Methods

### Ethics statement

All animal experiments were conducted in compliance with institutional guidelines of Animal experiment center of Wuhan University/Animal Biosafety Level-III laboratory for the Use of Animals. All animal procedures were approved by Wuhan University School of Medicine Animal Care and Use Committee.

### Mouse breast tumor-bearing model

Six-to-eight week old wild type BALB/c mice were purchased from Animal experiment center of Wuhan University/Animal Biosafety Level-III laboratory (Wuhan, China). Parental 4T1 cells or 4T1/DOX cells were intravenously inoculated into BALB/c mice through the tail vein. And in some cases, 4T1 breast tumor-bearing mice were repeatedly injected with DOX (5 mg/kg) or PBS at 15 days post-tumor implantation; 4T1/DOX breast tumor-bearing mice were treated with/without Compound C treatment (25 mg/kg, one time/every three days for four times). Two weeks later, the mice were sacrificed by inhalation of carbon dioxide for an average of 5 min, the percentage of M-MDSCs and G-MDSCs and the expression of miR-10a of MDSCs (cells were gated on CD11b^+^Gr-1^+^) in the spleen were detected.

### Cell culture

Mammary adenocarcinoma cancer 4T1 cells were grown in DMEM (Gibco) supplemented with 10% fetal calf serum (FCS) and 2 mM glutamine (Gibco) at 37 °C under 5% CO_2_. 4T1 cells were treated with 50 nM doxorubicin (doxorubicin hydrochloride, Sigma) for 72 hours when cells reached a confluency of 80%. After treatment doxorubicin containing medium was replaced by fresh medium. As soon as cells recovered, they were seeded for the next treatment cycle. In this manner, four rounds of treatment were performed to obtain the doxorubicin resistant 4T1 cells (4T1/DOX).

### Culture of mouse BM cells

Tibias and femurs bones from BALB/c mice were removed using sterile techniques and bone marrow (BM) was flushed. Red blood cells (RBCs) were lysed with ammonium chloride. To obtain BM-derived MDSCs, 2.5×10^6^ cells were plated into dishes with 100 mm diameter (Falcon, BD, NJ, USA) in 10 ml of medium supplemented with 10% (v/v) irradiated fetal bovine serum, 1 mM Sodium Pyruvate, 50 μM β-mercaptoethanol, 100 U/ml Penicillin, 150 U/ml Streptomycin and 2 mM L-glutamine, in a 5% CO_2_ and 37 °C incubator. BM cells were treated with 20 ng/ml of GM-CSF and 40 ng/ml of IL-6 (both from ebioscience) for 4 days. In some cases, PGE_2_ (100 μM, Cayman Chemicals), PKA-specific cAMP analog 6-Bnz-cAMP (500 μM), AH6809 (10 mΜ, Sigma-Aldrich), EP4 antagonist (ONO-AE3-208, Sigma-Aldrich), metformin (10 mM, Sigma-Aldrich) and/or Compound C (5 μM, Sigma-Aldrich) were added at the beginning of the 5-day culture. Cells were assessed by flow cytometry, or were used for RNA analysis by Real-time PCR.

### RNA extraction and quantitative RT-PCR

Total RNA from MDSCs or 4T1 tumor cells was isolated using the miRNeasy Mini Kit (Qiagen) according to the manufacturer’s instructions and was reverse transcribed to cDNA using miScript II RT kit (Qiagen). Quantitative RT-PCR (qRT-CPR) analyses for miR-10a and U6 (used as a normalization control) were performed using SYBR Green Master Mix and primers obtained from Qiagen. For assessing expression of IL-12, NOS2, TNF-α, Arg1, MMP9, and GAPDH, RNA (1 μg) was reverse-transcribed with Superscript III and random primers (Invitrogen). cDNA samples were amplified in a CFX96 Real-time System (Bio-Rad Laboratories, Hercules, CA, USA) and SYBR Green Master Mix (Invitrogen) and specific primers (Supplementary Table S1) according to the manufacturer’s instructions. All primers were purchased from Eurofins MWG Operon. Fold changes in mRNA expression between treatments and controls were determined by the δCT method. The data were normalized to a GAPDH reference.

### Cell transfection of miR-10a and its inhibitor

BM cells were cultured on 6-well plates and transfected in the following day. For miRNA knockdown, 100 pmol miR-10a antagomir was used. Oligonucleotides with random sequence served as negative controls for miRNA agomirs or antagomirs.

### Flow cytometry

Fluorescence-activated cell sorting (FACS) analyses on mouse cells were performed using PE-conjugated antibodies to Gr-1 (RB6-8C5), PE-conjugated antibodies to CD11c (N418), and allophycocyanin (APC)-conjugated antibodies to CD11b (M1/70). All antibodies were purchased from ebioscience and diluted to 1:100.

### PGE_2_, ATP, IFN-γ and IL-10 assays

The level of prostaglandin E_2_ released from 4T1 cells was measured using Prostaglandin E_2_ EIA (Cayman Chemicals). ATP in cell culture supernatants was measured using an ATP determination kit (Molecular Probes). IFN-γ and IL-10 in culture supernatants was quantified using ELISA kits (eBioscience). The assays were carried out according to the manufacturer’s instructions.

### MDSCs suppression assay

CD11b^+^Gr-1^+^ MDSCs were purified using magnetic microbeads (Miltenyi Biotec) or from FACS sorting. CD4-depleted mouse splenocytes treated with Mitomycin C (Sigma) were used as APCs. For CFSE (Invitrogen) dilution analysis, CD4^+^CD25^−^ naive T cells directly purified from mouse spleen and labeled with 5 μM CFSE for 10 min at 37 °C in PBS containing 0.1% BSA and washed twice with complete RPMI 1640 medium. This assay was conducted in round bottom 96 well plates with an addition of 5 μg/ml of anti-CD3 (eBiosciences) in 200 μl. CD4^+^: MDSC ratio was titrated and cells or supernatant were collected after 3–5 days of culture for FACS or ELISA analysis.

### Western blotting

Cells were lysed in protein lysis buffer containing 1% Triton X-100, 0.1% SDS, 150 mM NaCl, 50 mM Tris-HCl, 1 mM EDTA, 1 mM EGTA, 5 mM sodium molybdate, and 20 mM phenylphosphate with protease and phosphatase inhibitors (1 mM PMSF, 10 μg/ml aprotinin, 20 μg/ml leupeptin, 20 μg/ml pepstatin A, 50 mM NaF, and 1 mM sodium orthovanadate). Proteins of lysed cells were separated on 10% polyacrylamide gels using SDS-PAGE and transferred to nitrocellulose membranes. The membranes were stained with Ponceau Red to validate that all samples contained similar amounts of protein.

### Statistical analysis

All data are presented as mean ± SD and analyzed by using Microsoft Excel software (Version 2013). Comparison between two groups for statistical significance were performed with unpaired Student’s t test. For more groups, one-way ANOVA followed by Neuman-Keuls post hoc test was used. *p* < 0.05 was considered statistically significant.

## Additional Information

**How to cite this article**: Rong, Y. *et al*. Doxorubicin resistant cancer cells activate myeloid-derived suppressor cells by releasing PGE_2_. *Sci. Rep.* **6**, 23824; doi: 10.1038/srep23824 (2016).

## Supplementary Material

Supplementary Information
