# Supplementary material for: Doxorubicin resistant cancer cells activate myeloid-derived suppressor cells by releasing PGE2
Source: Sci Rep. 2016 Apr 1;6:23824. doi: 10.1038/srep23824 (PMC4817121; doi:10.1038/srep23824)
Supplement: Supplementary Information [file srep23824-s1.pdf]

Supporting information

Doxorubicin resistant cancer cells activate myeloid-derived suppressor cells by releasing PGE_2

Yuan Rong[1], Chun-Hui Yuan[2], Zhen Qu[2], Hu Zhou[1], Qing Guan[2], Na Yang[1], Xiao-Hua Leng[3], Lang Bu[4], Ke Wu[5], Fu-Bing Wang[1]*

[1] Department of Laboratory Medicine & Center for Gene Diagnosis, Zhongnan Hospital of Wuhan University, No. 169 Donghu Road, Wuchang District, Wuhan 430071, P.R. China

[2] Department of Immunology, School of Basic Medical Sciences, Wuhan University, No. 185 Donghu Road, Wuchang District, Wuhan 430071, P.R. China

[3] Hubei Key Laboratory of Tumor Biological Behaviors & Hubei Cancer Clinical Study Center, Zhongnan Hospital of Wuhan University, No. 169 Donghu Road, Wuchang District, Wuhan 430071, P.R. China

[4] Department of Pathophysiology, School of Basic Medical Sciences, Wuhan University, No. 185 Donghu Road, Wuchang District, Wuhan 430071, P.R. China

[5] Animal experiment center of wuhan university/Animal Biosafety Level-III laboratory, No. 115 Donghu Road, Wuchang District, Wuhan 430071, P.R. China

Corresponding author: Fu-Bing Wang, M.D., Ph.D., Department of Laboratory Medicine, Zhongnan Hospital of Wuhan University, No. 169 Donghu Road, Wuchang District, Wuhan 430071, P.R. China. Tel: +86-27-67813517; Fax: +86-27-67813128; Email address: wfb20042002@sina.com

Supplementary Table S1. Primers used for Real-time PCR

**Gene name**	**Forward primer**	**Reverse primer**
NOS2	CCAAGCCCTCACCTACTTCC	CTCTGAGGGCTGACACAAGG
Arg-1	CTCCAAGCCAAAGTCCTTAGAG	AGGAGCTGTCATTAGGGACATC
GAPDH	AGGTCATCCCAGAGCTGAACG	ACCCTGTTGCTGTAGCCGTAT
β-Actin	ACGGCCAGGTCATCACTATTC	AGGAAGGCTGGAAAAGAGCC
TNF-α	TCTATGGCCCAGACCCTCAC	GACGGCAGAGAGGAGGTTGA
COX2	TGAGTACCGCAAACGCTTCT	CTCCCCAAAGATAGCATCTGG
MMP9	TGAGCTGGACAGCCAGACACTAAA	TCGCGGCAAGTCTTCAGAGTAGTT

Supplementary Figures

Supplementary Figure S1

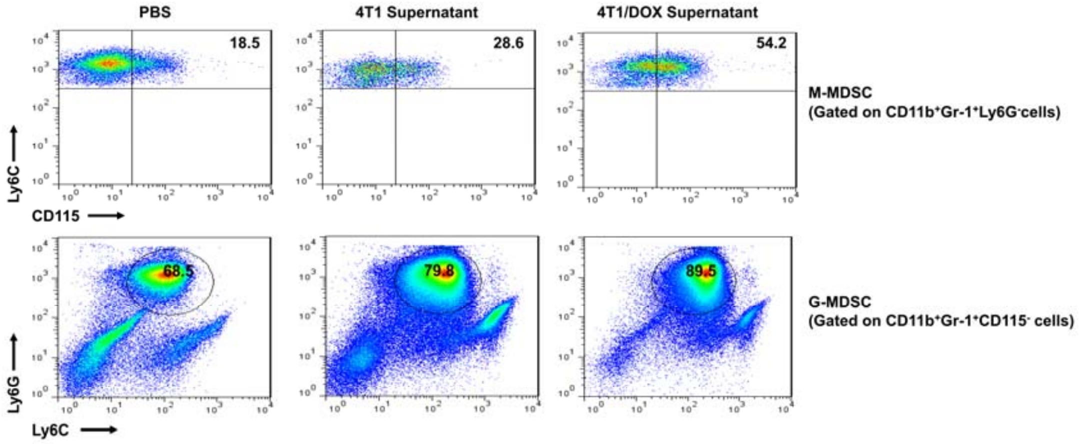

Supplementary Figure S1. Bone marrow (BM) cells were cultured with PBS or conditioned medium from 4T1 or Doxorubicin-resistant 4T1 cells (4T1/DOX) for 4 days in the presence of GM-CSF and IL-6, the frequency of M-MDSCs and G-MDSCs in the BM cells was analyzed by flowcytometry.

Supplementary Figure S2

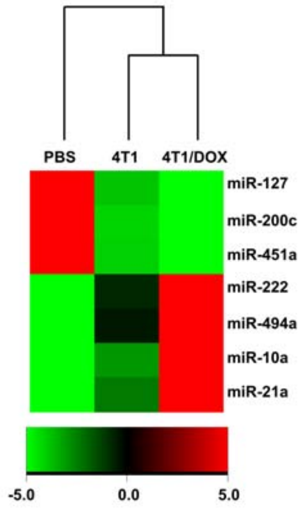

Supplementary Figure S2. MicroRNA expression profiles in MDSCs treated by PBS, Supernatant from 4T1 or 4T1/DOX.

Supplementary Figure S3

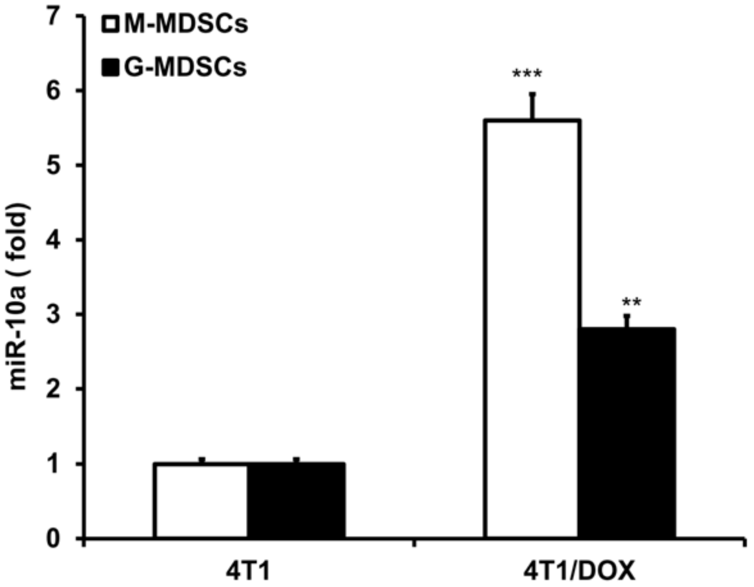

Supplementary Figure S3. BM cells were cultured with 4T1 cells and 4T1/DOX cells-derived conditioned medium; in the presence of GM-CSF and IL-6 for 24 h, and the expression of miR-10a in M-MDSCs or G-MDSCs was measured by qRT-PCR. The data represent Mean ± SD, (n=5). ** $p< 0.01$, ***$p<0.0001$.

Supplementary Figure S4

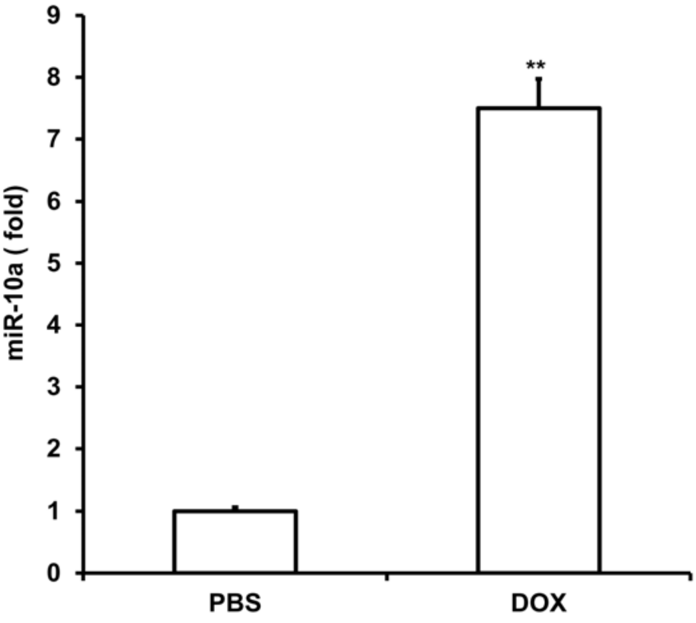

Supplementary Figure S4. The expression of miR-10a in MDSCs sorted from tumor xenograft of 4T1 tumor bearing mice receiving repeated injections of doxorubicin (5mg/kg) or PBS was analyzed by qRT-PCR. The data represent Mean ± SD, (n=5). ** $p < 0.01$.
